# Face mask reduces gaze-cueing effect

**DOI:** 10.1038/s41598-023-40195-5

**Published:** 2023-08-12

**Authors:** Han Jia, Qi Wang, Xinghe Feng, Zhonghua Hu

**Affiliations:** Institute of Brain and Psychological Sciences, Sichuan Normal University, Chengdu, 610068 People’s Republic of China

**Keywords:** Neuroscience, Psychology

## Abstract

Recent studies have found that face masks affect social cognition and behaviour in the context of the novel coronavirus (COVID-19) pandemic. The eyes, the only part of the face not covered by face masks, are an important spatial attention cue that can trigger social attention orienting. Here, we adopted a spatial gaze-cueing task to investigate whether face masks affect social attention orienting triggered by eye gaze cues. In Experiment 1, participants were asked to determine the orientation of a target line under two types of cues—masked and non-masked faces—and two stimulus onset asynchrony (SOA) conditions (300 ms and 1000 ms). The results showed that masked faces induced a smaller gaze-cueing effect (GCE) compared to non-masked faces at 300 ms SOA, while two face types induced similar GCEs at 1000 ms SOA. Experiment 2 used mouth-obscured faces and non-masked faces as cues and found that no significant difference in GCE between the two types at either 300 ms or 1000 ms SOA, indicating that the reduction of GCE caused by the masked face was due to the social meaning expressed by the mask rather than a physical effect of masking. The present study extends previous findings to support the idea that high-level social information affects the processing of eye gaze direction and provides evidence that face masks affect social cognition and behaviour in the context of COVID-19.

## Introduction

The outbreak of the novel coronavirus (COVID-19) made medical masks a necessity in people’s daily lives. The medical mask can reduce the likelihood of virus transmission by preventing the dispersion of saliva particles in the air^1^ and positively impacts epidemic prevention and control. Therefore, many countries and health organisations had promoted wearing masks as a critical strategy to prevent the spread of the novel coronavirus.

Human faces carry a large amount of social information, such as gender, identity, facial expressions, and gaze information^2–4^, which is indispensable in our daily social interactions. Since masks cover a large part of the face, they can affect the processing of facial information about the wearer^5^. Previous studies have found that wearing masks can affect individuals’ ability to infer the credibility of people through facial information^6^, impair recognition of faces^7^, and reduce emotional recognition^8^.

The eyes, as the exposed part of the face, provide gaze direction information of individuals wearing masks. Gaze direction indicates the location and object that captures one’s attention. By perceiving the gaze direction of others’ eyes, we can infer their behavioural intentions and psychological states^9^. Therefore, as an essential non-verbal cue, eye gaze direction information plays a vital role in social communication. Moreover, during social interaction, the shifting of one’s gaze direction may indicate interest sharing or hints of a potential imminent threat.

Observers often quickly follow the shifts in others’ gaze direction^10^. In real life, imagine that you are talking to a person and suddenly notice that the other person’s gaze shifts to an object on your left side. Will you be curious about the sudden shift in gaze and follow it to look at that object? If so, you have completed a shift of attention cued by the other person’s gaze direction. In experiments, researchers used an adapted Posner paradigm to confirm that others’ gaze direction, as a spatial cue, induced observers’ attention to shift^11^. They first presented a face that looked directly at the subject in the centre of the screen and then presented a face that looked to the left or right. After certain time intervals, a target was presented on either the left or right side of the screen. In other words, a target might appear either in the congruent location of the gaze cue or in an incongruent position. Participants were told to distinguish the target’s location and press the equivalent key. Studies found that the reaction time is significantly reduced when the targets are presented in the congruent location of the gaze cue^11–13^. This reduced effect of the gaze-at location is called the gaze-cueing effect (GCE).

Previous studies have found that the gaze-cueing effect is affected by context and facial features (such as facial expression, facial identity, etc.)^14^. Wearing a mask affects the observer’s facial information process^5^. Therefore, it is possible that viewing a masked face affects the cue effect induced by gaze direction.

On the one hand, recent studies have found that masks can affect social cognition. Participants believed that masked faces were more trustworthy and likeable than those without masks^6^. Faces with high trustworthiness induce a greater gaze-cueing effect than those with low trustworthiness^15,16^. In addition, masked faces may imply threatening scenes. For example, robbers wear masks or scarves when robbing or stealing, and doctors wear masks in hospitals when injecting patients. Studies have shown that threatening situations or threatening facial expressions affect the gaze-cueing effect^17–19^. Therefore, we speculate that the related social meaning induced by masked faces may also affect the gaze-cueing effect.

On the other hand, although wearing a mask covers some features of the face (e.g., mouth and nose), the eye, which carries gaze direction, is not covered. Previous studies have found that presenting only the eye region produces a gaze-cueing effect similar to presenting the entire face^20,21^. A possible explanation is that humans have evolved differently from other animals in terms of the proportion of sclera and iris. This solid graphic contrast makes human eyes more prominent on the face. Even without other facial information, gaze direction can still be perceived well through the eye region. Therefore, masked faces may not affect the gaze-cueing effect^22^.

It is worth noting that two studies have examined the modulation of individuals’ GCE when viewing faces wearing masks. In one of the studies, Dalmaso et al.^22^ used Italian and Chinese participants and found that masks did not significantly affect GCE. In another research, Villani et al.^42^ combined the gaze cue paradigm with the Simon task and found an interaction effect between gaze cue-target congruency, face masks, and the Simon effect, which was specifically reflected in the mask condition the GCE emerged only when target and response positions corresponded (i.e., Simon corresponding trials). Consistent with Dalmaso^22^, Villani et al.^42^ also did not show that face masks could alter GCE due to the absence of a two-way interaction effect (face masks × congruency). Combining these two studies, we found the face mask does not influence GCE. Nevertheless, it is important that the data collection time for the above studies all took place in the early years of COVID-19 (2020). However, it may take time for both behavioral responses and cognitive changes about face masks to better socially learn the meaning of face masks during COVID-19, and the perception of a person with a face mask is influenced by the historical period and the geographical region in which the study has been conducted. This means that the impact of masks on GCE may take time to be observed. Therefore, it is necessary to revisit this issue in the middle and late stages of COVID-19.

In addition, a larger number of studies have reported gender differences in gaze perception processing. In a modified Posner cueing paradigm, women were reported to have a greater gaze-cueing effect than men^18,23–27^. In a gaze direction judgement task, men reported more direct gaze than women^28,29^. Furthermore, women and men showed different patterns in the interaction between gaze direction perception and other factors, such as face familiarity^30^, facial expressions^31^ and oxytocin^32,33^. Thus, the gender of participants may be a potential moderating factor in exploring the effect of face masks on gaze-cueing effect.

Taken together, we assume that, in the middle and late stages of COVID-19, masks may impact facial cognition, which further affects the gaze-cueing effect. It is also possible that the mask does not cover the eyes, thus not affecting the gaze-cueing effect. Therefore, in this study, we used two types of faces, masked and non-masked, to explore the effect of masked faces on the gaze-cueing effect. In addition, to determine whether the effect of masked faces on the gaze-cueing effect is related to individuals’ cognition, the coronavirus, or individuals’ characteristics, we asked participants to fill out a four-item questionnaire on COVID-19, the Social Interaction Anxiety Scale (SIAS)^34^, the Social Phobia Scale (SPS)^34^, the Self-rating Depression Scale (SDS)^35^, and the Self-rating Anxiety Scale (SAS)^36^ after completing the gaze-cueing task. Finally, we also hypothesized that the effect of masks on gaze-cueing effect may be moderated by the gender of the participants.

## Experiment 1

### Methods

#### Participants

Data were collected from September 17, 2021, until October 11, 2021, during the regular prevention phase of COVID-19 in Chengdu, China. In particular, during the time window in which data were collected, the new daily cases of COVID-19 in China increased from 12 to 72, according to the National Health Commission of the People's Republic of China (http://www.nhc.gov.cn/xcs/yqtb/list_gzbd.shtml).

Forty-six students (23 men, mean age = 19.41, SD = 1.28) from Sichuan Normal University participated in this study. All participants were right-handed and had normal or corrected-to-normal vision. We calculated the a priori power of the experiment using G*power 3.1^37^ to ensure it was adequately powered. We assumed a medium effect size (f = 0.25) based on theoretical considerations and set α = 0.05. According to G*power, we needed a sample size of at least 20 to obtain a significant interaction with an actual power of 0.911. In addition, the range of sample sizes was roughly 24–41 in previous studies on GCE^21,38,39^, and one study related to masks took a participant size of 46^22^, so we determined that the amount of participants was reasonable. This experiment was approved by the Ethics Committee of Sichuan Normal University [SCNU-210304]. The study was carried out in accordance with the norms of the Declaration of Helsinki, and all participants signed informed consent forms prior to their participation.

#### Stimuli

**Figure 1:**
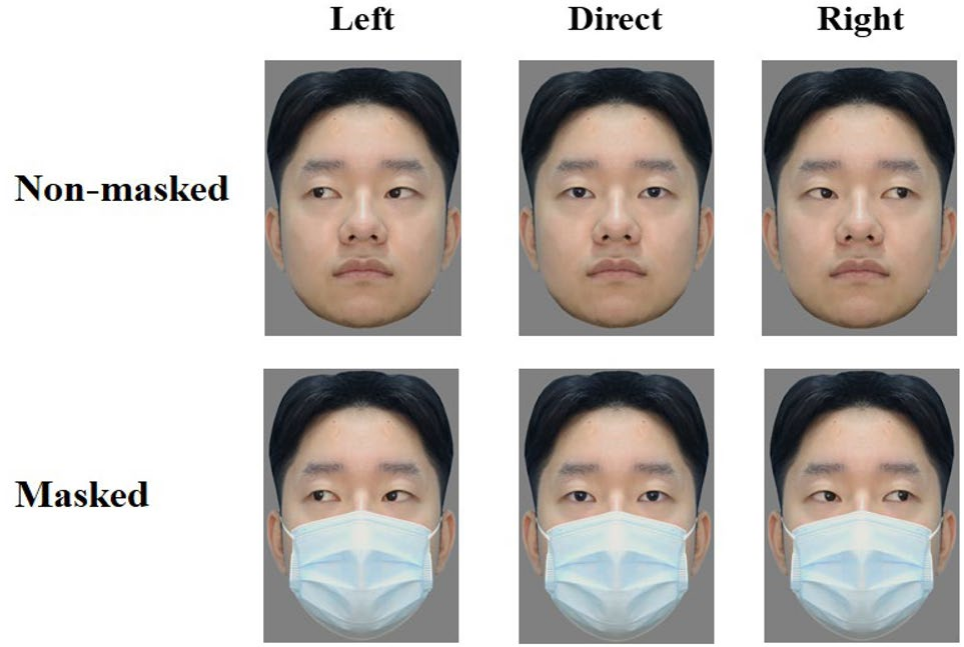
Three gaze directions (left, direct, right) with and without mask. Written informed consent was obtained from the participants for publication of their facial images in the academic journal.

We used a set of 36 facial images, including six face identities (three males) with a neutral expression. Each identity displays a direct gaze (straight towards the camera), a gaze averted 30° to the left of the camera, and a gaze averted 30° to the right of the camera, with and without mask (see Fig. 1). Hence, there were six images of each facial identity. All images were adjusted in brightness, contrast, and resolution (283 pixel width × 389 pixel height, 5.7° × 6.8°) with Photoshop CS6. All stimuli were presented using E-prime 2.0, centred on a gray background, and reproduced on a 23.8-in ThinkVision TE24-10 monitor.

#### Procedure

**Figure 2:**
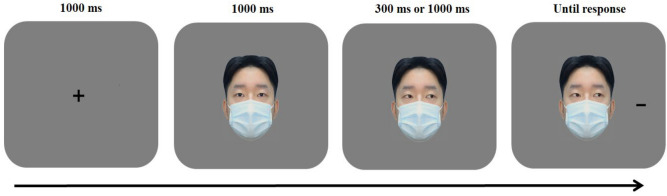
Sequence of events in a single trial.

Participants were instructed to sit comfortably in an acoustically shielded room at a distance of 60 cm from the monitor. They completed one practice session of 12 trials before the experimental gaze-cueing task for the study, which consisted of 6 blocks of 64 trials. Each experimental condition was selected randomly and presented equally. In a single trial, a fixation cross appeared at the centre of the screen for 1000 ms followed by a central face with direct gaze which appeared for 1000 ms. Then, a central face with averted gaze appeared for either 300 ms or 1000 ms (stimulus onset asynchrony (SOA)). Next, a black target line (2.7° × 0.2° or 0.2° × 2.7°) appeared either leftwards or rightwards (11.3°) until response (see Fig. 2). Participants were asked to determine the orientation of the target line by pressing “1” or “2” on the keyboard. They were also instructed to respond as quickly and as accurately as possible. Feedback on accuracy, presented only in the practice task, appeared on the screen (i.e., the word “correct” in the colour blue or “wrong” in the colour red) after every response. After completing the formal task, participants were asked to fill in the self-reported survey in Chinese, including a Self-Rating Anxiety Scale (SAS)^36^, a Social Phobia Scale (SPS)^34^, a Social Interaction Anxiety Scale (SIAS)^34^, a Self-Rating Depression Scale (SDS)^35^, and four questions assessing COVID-related habits and attitudes (see Supplementary information).

### Data analysis

A repeated-measures analysis of variance (ANOVA), with cue–target congruency (congruent vs. incongruent), face type (masked vs. non-masked), and SOA (300 ms vs. 1000 ms) as within-subjects factors and the gender of the participant (men vs. women) as a between-subjects factor, was performed on accuracy and RT. The results did not reveal a main effect of gender or interaction between gender and face type or a four-way interaction effect, so we conducted a three-way ANOVA, with cue–target congruency (congruent vs. incongruent), face type (masked vs. non-masked), and SOA (300 ms vs. 1000 ms) as within-subjects factors, was performed on accuracy and RT, and report the results of the 3-way ANOVA only.

To further explain the three-way interaction, we calculated the gaze-cueing effect (RT_incongruent_ − RT_congruent_) in each face type × SOA condition. A repeated-measures ANOVA, with SOA and mask condition as within-subject factors, was performed on GCE.

### Results and discussion

#### Accuracy

The analysis revealed a main effect of SOA, *F*(1, 45) = 14.990, *p* < 0.001, ${\eta }_{p}^{2}$ = 0.250, with more accurate responses in 1000 ms SOA compared with responses in 300 ms SOA. There was also a two-way interaction between cue–target congruency and SOA, *F*(1, 45) = 7.908, *p* = 0.007, ${\eta }_{p}^{2}$ = 0.149, with more accurate responses in the congruent condition than in the incongruent condition only in 300 ms SOA (*p* = 0.003) but not in 1000 ms SOA (*p* = 0.212). No other significant main or interaction effects were found in RTs, *ps* > 0.05 (see Table 1).

**Table 1:** Accuracy (*M* ± *SD*) and reaction times (*M* ± *SD*, ms) for cue–target congruency, face type, and SOA in Experiment 1.

		Masked face		Non-masked face	
		300 ms	1000 ms	300 ms	1000 ms
**Congruent**	ACC	0.97 ± 0.03	0.96 ± 0.04	0.96 ± 0.04	0.97 ± 0.03
	RT	682.78 ± 140.71	647.65 ± 125.04	670.93 ± 145.32	658.96 ± 141.03
**Incongruent**	ACC	0.95 ± 0.04	0.97 ± 0.03	0.95 ± 0.03	0.97 ± 0.04
	RT	689.23 ± 134.31	668.40 ± 143.19	701.34 ± 156.15	670.14 ± 140.38

#### Reaction time

**Figure 3:**
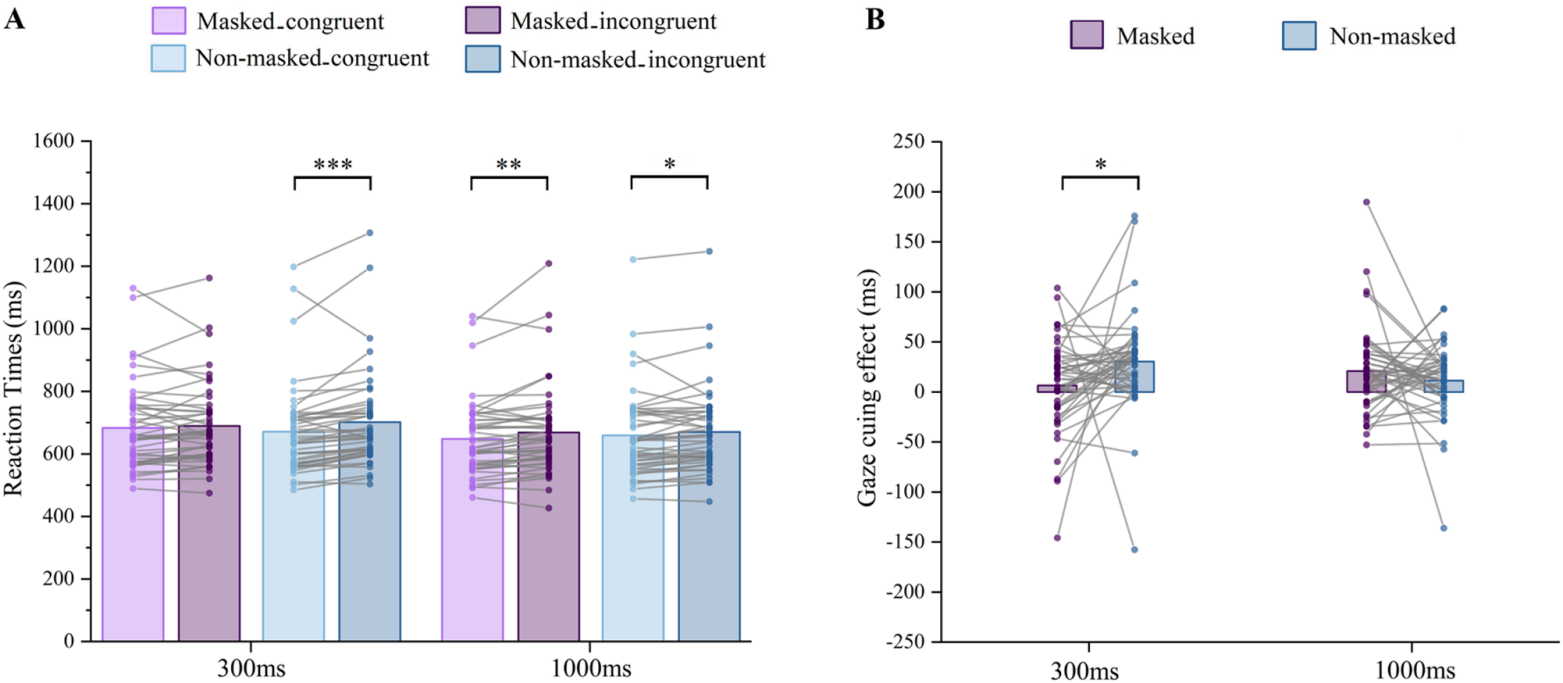
(**A**) The mean reaction times for SOA × congruency × face type; (**B**) the gaze-cueing effect for masked and non-masked faces with SOA 300 ms and SOA 1000 ms. The dots (●) indicate data for each participant, *p < 0.05, **p < 0.01, ***p < 0.001.

Incorrect responses (3.73%) and outliers (1.99%)—response times outside 3 standard deviations—were excluded from the RT analysis. The analysis showed that SOA had a significant main effect, *F*(1, 45) = 31.517,* p* < 0.001, ${\eta }_{p}^{2}$ = 0.412, with faster responses in 1000 ms SOA than in 300 ms SOA, as did the main effect of cue–target congruency, *F*(1, 45) = 34.541,* p* < 0.001, ${\eta }_{p}^{2}$ = 0.434, with faster responses in the congruent condition than in the incongruent condition. Moreover, the three-way interaction between face type, cue–target congruency, and SOA was significant: *F*(1, 45) = 7.700, *p* = 0.008, ${\eta }_{p}^{2}$ = 0.146. The simple effect analysis showed an absence of cue–target congruency effect of the masked face in the 300 ms SOA (*p* = 0.355), while the RTs of the congruent condition were significantly faster than in the incongruent condition for non-masked faces in the 300 ms SOA (*p* < 0.001), as well as in the 1000 ms SOA, which produced significant gaze-cueing effects for both masked (*p* = 0.002) and non-masked faces (*p* = 0.043) (see Fig. 3A). No other significant main or interaction effects were found in RTs, *ps* > 0.05 (see Table 1).

#### Gaze-cueing effect

The analysis on GCE showed that the interaction between SOA and face type reached significance, *F*(1, 45) = 7.700, *p* = 0.008, ${\eta }_{p}^{2}$ = 0.146, with smaller GCE for masked faces than non-masked faces in 300 ms SOA (*p* = 0.038), while there was no significant difference in 1000 ms SOA (*p* = 0.289). In another direction, GCE for 300 ms SOA was greater than that for 1000 ms SOA for non-masked face (*p* = 0.027), while there was no such difference for masked faces (*p* = 0.093) (see Fig. 3B). No other significant main and interaction effects were found in GCE, *ps* > 0.05.

#### Correlations between GCE and Questionnaires

We calculated the total scores for SAS, SPS, SIAS, SDS, and a four-item questionnaire on COVID-19. Pearson correlation analysis was performed with the GCE of each face type condition (masked vs. non-masked) and the scores on each scale and questionnaire as variables. There were no significant correlations, *ps* > 0.05 (see Supplementary information).

In this experiment, we investigated whether observing masked faces resulted in a greater gaze-cueing effect than non-masked faces. Along with the robust gaze-cueing effect, we found an absence of cue effect of the masked face in 300 ms SOA. This finding implied a possible influence on an individual’s behaviour in perceiving gaze shifts of someone who is wearing a mask. Based on these results, viewing a masked face can have significant influences on the corresponding rapid shift of attention in observers at the short SOA (300 ms). We suggest that the social meaning conveyed by a face mask was responsible for the reduction of the gaze-cueing effect. For example, people wearing masks were likelier to signal meaningful information about themselves or the surrounding environment.

## Experiment 2

The results of Experiment 1 showed that a masked face triggers a reduced gaze-cueing effect. We believe that the reduction of GCE was related to the social meaning of a face mask. However, another possibility is that the absence of information from the lower facial features when obscured by a face mask hinders the perception of gaze cues and reduces GCE. A mask covering the mouth and nose area is likely to make individuals find it more difficult to perceive the gaze direction in a short SOA (300 ms). To rule out this possibility, we conducted Experiment 2. By cutting off the lower half of faces in the images, we created two types of face—mouth-obscured and non-masked—in Experiment 2. We posited that the lack of lower facial information was responsible for the decrease in GCE. In that case, we would observe a reduction of gaze-cueing effect for the mouth-obscured faces. Otherwise, we would find no significant differences between non-masked and mouth-obscured conditions in Experiment 2.

### Methods

#### Participants

Data were collected from November 1, 2021, until November 15, 2021, during the regular prevention phase of COVID-19 in Chengdu, China. In particular, during the time window in which data were collected, the new daily cases of COVID-19 in China increased from 22 to 109, according to the National Health Commission of the People's Republic of China (http://www.nhc.gov.cn/xcs/yqtb/list_gzbd.shtml).

Forty-six students (23 men, mean age = 19.11, SD = 1.45), different from Experiment 1, from Sichuan Normal University participated in this study. All participants were right-handed and had normal or corrected-to-normal vision. The experiment was approved by the Ethics Committee of Sichuan Normal University and carried out in accordance with the norms of the Declaration of Helsinki. All participants signed informed consent forms prior to their participation.

#### Stimuli

**Figure 4:**
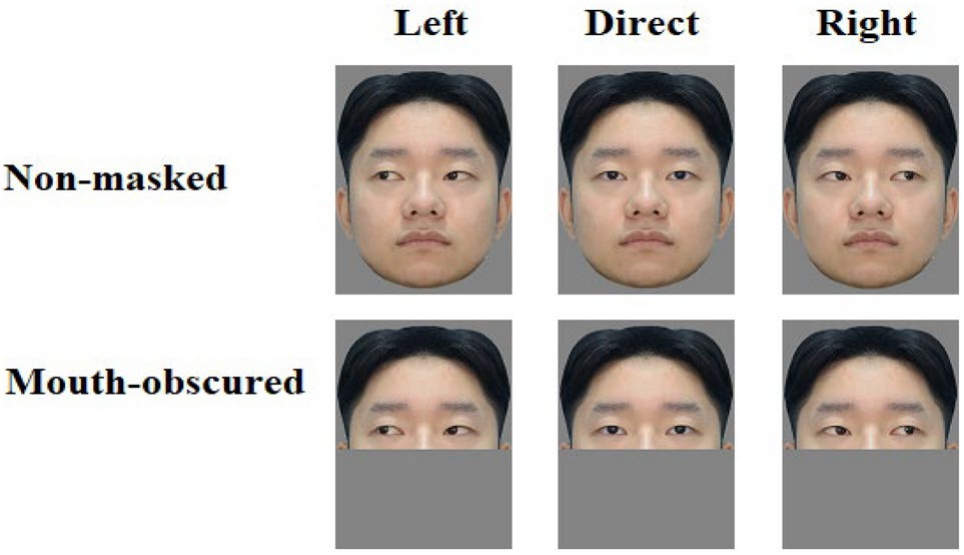
Three gaze directions (left, direct, right) for non-masked and mouth-obscured faces. The owners of example facial images consented to their portraits being published in any academic journal.

The facial identities used in Experiment 2 were the same as those in Experiment 1. The same facial images were presented without a mask and with the lower half of the image removed (see Fig. 4).

#### Procedure

The same procedures followed in Experiment 1 were repeated for Experiment 2.

### Data analysis

Data analysis was the same as in Experiment 1.

### Results and discussion

#### Accuracy

The analysis showed a significant interaction effect between cue–target congruency and SOA, *F*(1, 45) = 4.195, *p* = 0.046, ${\eta }_{p}^{2}$ = 0.085, with more accurate responses in 1000 ms SOA than in 300 ms SOA of cue–target incongruent condition (*p* = 0.022), while there was no difference in congruent conditions (*p* = 1). No other significant main and interaction effects were found in accuracy, *ps* > 0.05 (see Table 2).

**Table 2:** Accuracy (*M* ± *SD*) and reaction times (*M* ± *SD*, ms) for cue–target congruency, face type, SOA in Experiment 2.

		Mouth-obscured		Non-masked	
		300 ms	1000 ms	300 ms	1000 ms
**Congruent**	ACC	0.97 ± 0.03	0.97 ± 0.03	0.97 ± 0.03	0.97 ± 0.04
	RT	642.83 ± 104.53	613.14 ± 98.56	648.27 ± 111.39	613.92 ± 103.83
**Incongruent**	ACC	0.96 ± 0.04	0.97 ± 0.04	0.97 ± 0.03	0.98 ± 0.03
	RT	660.88 ± 102.84	629.37 ± 102.33	662.78 ± 103.54	637.51 ± 106.23

#### Reaction time

Incorrect responses (2.98%) and outliers (1.75%)—reaction times outside 3 standard deviations—were excluded from the RT analysis.

**Figure 5:**
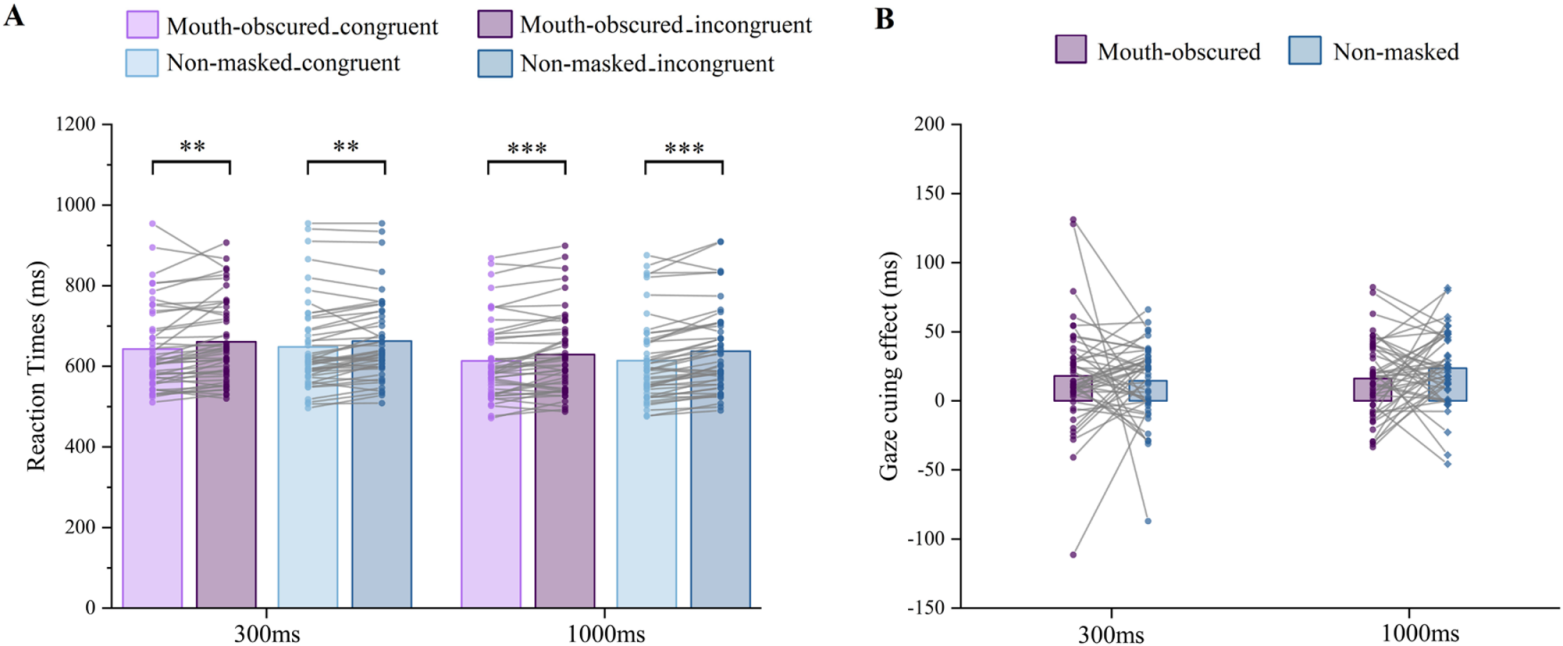
(**A**) Mean reaction time for SOA × congruency × face type. (**B**) gaze-cueing effect for obscured mouth and non-masked faces with SOA 300 ms and 1000 ms. The dot (filled circle) indicates data for each participant. **p < 0.01, ***p < 0.001.

The results showed a significant main effect of SOA, *F*(1, 45) = 74.192,* p* < 0.001, ${\upeta }_{\mathrm{p}}^{2}$ = 0.622, with faster responses in 1000 ms SOA condition than those in 300 ms SOA condition (see Fig. 5A). Likewise, the main effect of cue–target congruency, *F*(1, 45) = 58.690,* p* < 0.001, ${\eta }_{p}^{2}$ = 0.566, reported faster responses in the cue–target congruent condition than in the incongruent condition (see Fig. 5A). No other significant main or interaction effects were found in RTs, *ps* > 0.05 (see Table 2).

#### Gaze-cueing effect

For GCE, we did not find main effects of face type (*p* = 0.652) and SOA (*p* = 0.415). Moreover, the interaction between SOA and face type did not reach significance (*p* = 0.257) (see Fig. 5B).

### Correlations between GCE and Questionnaires

We calculated the total score for SAS, SPS, SIAS, SDS and four self-designed questions. Pearson correlation analysis, with GCE of each facial condition (mouth-obscured faces vs. non-masked faces) and the score of each scale and question as variables, was performed. We found no significant correlations, *ps* > 0.05 (see Supplementary information).

## Discussion

The current study used the spatial gaze-cueing task to investigate the modulation of individuals’ GCE when viewing faces wearing masks in the middle and late stages of COVID-19. We used masked and non-masked faces as stimuli in Experiment 1, and faces without masks and faces with the lower half removed (mouth-obscured faces) as stimuli in Experiment 2. The results showed that masked faces induced a smaller GCE in the 300 ms SOA condition, but mouth-obscured faces did not produce a GCE reduction effect similar to masked faces. Besides, we did not find a main effect of gender or interaction effect between gender and face type was significant in either Experiment 1 or Experiment 2. These findings showed that the observed face mask modulation was associated with the special social meaning of a face mask, rather than low-level physical differences between masked and non-masked faces and the effect of masks on GCE was not moderated by the gender of participants.

The current study provides new evidence that the perception processing of gaze direction can be modulated by face masks. However, the findings of our study were not entirely in line with the results of Villani et al.^42^, which reported that the GCE was increased by face masks when stimuli–response correspondence was taken into account. Conversely, the finding of our study showed that the GCE was decreased by face masks. This inconsistency may be due to the cultural diversity of the participants. The population of participants in Villani et al.^42^ was Italian, while the participants used in this study were Chinese. In the Chinese cultural context, it is considered more in the collective interests to wear a mask at the time of COVID-19, and such socially normative behavior is more likely to lead to a lower GCE^44^.

But, the cultural context alone does not fully explain our results. Because Dalmaso et al.^22^ collected data in both Italy and Guangdong, China, it was found that face masks did not alter the gaze-cueing effect for either Italians or Chinese. This suggests that the reasons for the divergence between this study and previous studies^22,42^ may be related to factors other than cultural background. First, the experimental settings are different between our study and Dalmaso et al.^22^. For example, limitations on response times and response methods are different. In the present study, the reaction time was unlimited, while Dalmaso et al.^22^ required participants to respond within 2000 ms. Besides, while both studies use the discrimination task, Dalmasso et al.^22^ needed 2 hands (f and k) while our study just need one right hand to make the response. This may have contributed to some elements of Simon effects in their study that were not present in our study. A direct comparison between the two studies is not appropriate due to the differences in experimental settings. In addition to the experimental setup, more importantly, the timing of the data collection is different between this study and previous studies^22,42^. The data collection for this study took place from September 17, 2021 to October 11, 2021 (Experiment 1), and November 1, 2021 to November 15, 2021(Experiment 2), respectively, whereas, in Dalmasso et al.^22^, the data collection took place on October 23, 2020 to November 7, 2020 (Experiment 1), and November 11, 2020 to November 13, 2020 (Experiment 2); in Villani et al. study^42^, data were collected from June 19, 2020, until December 8, 2020. Apparently, our study occurred approximately 2 years after COVID-19 and one year after previous studies^22,42^. This difference in the timing of data collection may have possibly resulted in different levels of social learning and familiarity with the face masks among the participants, resulting in differences in our results and previous studies^22,42^.

The findings of our study showed that social attention orienting triggered by gaze can be modulated by face masks, which is in line with previous studies showing that face masks affect judgment of gaze directions^40,41^. One possible explanation for the reduction of GCE triggered by masked faces is that wearing masks is considered a pro-social behaviour while wearing no mask is considered antisocial behaviour. Therefore, masked faces attract less social attention than faces without masks. Gaze cues are essential when inferring other people’s goals and expected behaviours in social interaction. Therefore, when interacting with individuals who blatantly violate social and behavioural norms, people feel the need to observe and follow their gaze behaviour more closely. Since the outbreak of COVID-19, wearing masks has become a primary means of preventing the spread of the virus among citizens. Faces wearing masks may be considered safer and more reliable than those without masks^43^. Wearing masks has become our social norm, a common feature in our daily lives, and even mandatory in some countries. Faces associated with socially normative behaviour, have been reported to induce a lower GCE than those judged to be engaging in antisocial behaviour^44^. Masked faces may be considered to follow social rules. Thus, the gaze shift of masked faces may be perceived as less likely to forecast potential risk or as a less valuable signal.

Additionally, the reduction of GCE for masked faces was observed only in 300 ms SOA, suggesting that the modulation of high-level social information on GCE occurs in the early stage of processing, which is consistent with previous studies^45–47^. Notably, a considerable number of studies have found that the modulation of high-level social information on GCE occurs not only under short SOA conditions, but also persists under long SOA conditions^44,48–52^. Thus, our results showed that the extraction of social information from face masks could be a fast and automatic process. This might reflect the evolutionary and social importance of masks as a form of face covering, but the effect does not last long.

Finally, there are some limitations to our study. First, we found no significant correlation between the scores of questionnaires (SAS, SDS, SIAS, SPS, and the COVID-19 questionnaire) and GCE in either experiment. This may be related to the small sample size of our study. Future research could use larger sample sizes or meta-analysis methods to further explore the correlation between personality traits, masks, and gaze direction processing. Second, we used Chinese people as participants, and Chinese faces in the experimental materials. Since the Chinese COVID-19 rules and prevailing collectivism, there has been greater acceptance and adherence to wearing a mask; recruiting participants from other regions may yield distinct results.

In conclusion, the present study provides preliminary evidence that social attention orienting triggered by gaze may be modulated by face masks. The results of our study showed that faces wearing masks triggered a smaller GCE, which may be related to the social meaning of masked faces rather than to the physical differences between masked and non-masked faces. In addition, the reduction in GCE associated with the face mask occurs in the early stage of processing and does not last long. Our findings are significant for understanding the impact of wearing masks on social cognition and behaviour during the COVID-19 pandemic.

### Supplementary Information

Supplementary Information.

## Data Availability

The data that support the findings of this study are available at https://data.mendeley.com/datasets/zfd3s5hpzn/1.
