# Supplementary material for: Face mask reduces gaze-cueing effect
Source: Sci Rep. 2023 Aug 12;13:13160. doi: 10.1038/s41598-023-40195-5 (PMC10423210; doi:10.1038/s41598-023-40195-5)
Supplement: Supplementary file 1 — Supplementary Information. [file 41598_2023_40195_MOESM1_ESM.docx]

Supplementary information for

**Face Mask Reduces Gaze-Cueing Effect**

Han Jia^1^ **^#^**, Qi Wang^1^ **^#^**, Xinghe Feng^1^**^#^**, Zhonghua Hu^1^

1. Institute of Brain and Psychological Sciences, Sichuan Normal University, Chengdu, PR China

Correspondence to:

Zhonghua Hu, Ph.D. (ORCID: 0000-0002-9213-457X)

Institute of Brain and Psychological Sciences, Sichuan Normal University, Chengdu, 610068, PR China, E-mail: [huzhonghua2000@163.com](mailto:huzhonghua2000@163.com)

**Note:** Han Jia, Qi Wang and Xinghe Feng have contributed equally to this study.

Table S1

*Correlation coefficients for questionnaire results analysis in Experiment 1 and 2*

|  | Experiment 1 | | Experiment 2 | |
| --- | --- | --- | --- | --- |
|  | Masked GCE | Non-Masked GCE | Mouth-obscured GCE | Non-Masked GCE |
| Q1^a^ | 0.160 | -0.240 | -0.020 | 0.087 |
| Q2^b^ | 0.099 | -0.295 | -0.159 | -0.090 |
| Q3^c^ | -0.012 | -0.077 | -0.218 | 0.038 |
| Q4^d^ | 0.207 | -0.060 | -0.228 | 0.128 |
| SAS^e^ | 0.123 | 0.178 | -0.226 | -0.004 |
| SPS^f^ | 0.177 | -0.025 | -0.162 | 0.072 |
| SIAS^g^ | 0.151 | 0.035 | -0.163 | 0.002 |
| SDS^h^ | 0.208 | 0.201 | -0.078 | -0.095 |

**Note**: The significance of the correlation coefficients was corrected by multiple comparisons, and there were no significant results

(a) Q1 = I wear a mask when I go out during the COVID-19 pandemic;

(b) Q2 = I think masks played a big role in protection during the COVID-19 pandemic;

(c) Q3 = In public, I prefer to communicate with people wearing masks;

(d) Q4 = I don't think wearing masks will affect my social activities with others;

(e) SAS = Self-Rating Anxiety Scale;

(f) SPS = Self-Rating Depression Scale;

(g) SIAS = Social Interaction Anxiety Scale;

(h) SDS = Self-Rating Depression Scale.
